# Gas6 and the Receptor Tyrosine Kinase Axl in Clear Cell Renal Cell Carcinoma

**DOI:** 10.1371/journal.pone.0007575

**Published:** 2009-10-30

**Authors:** Anna Gustafsson, Anna-Karin Boström, Börje Ljungberg, Håkan Axelson, Björn Dahlbäck

**Affiliations:** 1 Department of Laboratory Medicine, Section for Clinical Chemistry, University Hospital Malmö, Lund University, Malmö, Sweden; 2 Department of Laboratory Medicine, Center for Molecular Pathology, University Hospital Malmö, Lund University, Malmö, Sweden; 3 Department of Surgical and Perioperative Sciences, Urology and Andrology, Umeå University, Umeå, Sweden; Health Canada, Canada

## Abstract

**Background:**

The molecular biology of renal cell carcinoma (RCC) is complex and not fully understood. We have recently found that the expression of the receptor tyrosine kinase Axl in the RCC tumors independently correlates with survival of the patients.

**Principal Findings:**

Here, we have investigated the role of Axl and its ligand Gas6, the vitamin-K dependent protein product of the *growth arrest-specific gene 6*, in clear cell RCC (ccRCC) derived cells. The Axl protein was highly expressed in ccRCC cells deficient in functional von Hippel-Lindau (VHL) protein, a tumor suppressor gene often inactivated in ccRCC. VHL reconstituted cells expressed decreased levels of Axl protein, but not Axl mRNA, suggesting VHL to regulate Axl expression. Gas6-mediated activation of Axl in ccRCC cells resulted in Axl phosphorylation, receptor down-regulation, decreased cell-viability and migratory capacity. No effects of the Gas6/Axl system could be detected on invasion. Moreover, in ccRCC tumor tissues, Axl was phosphorylated and Gas6 γ-carboxylated, suggesting these molecules to be active *in vivo*.

**Significance:**

These results provide novel information regarding the complex function of the Gas6/Axl system in ccRCC.

## Introduction

The receptor tyrosine kinases (RTKs) constitute a superfamily of transmembrane proteins that relays signals from extracellular growth factors into the cell. [1], [2]

The TAM subfamily of RTKs contains the receptors Axl, Tyro3, and Mer [3], [4]. They have in common a unique extracellular domain composed of two N-terminal immunoglobulin-like domains and two fibronectin type III repeats similar to the structure of neural cell adhesion molecules (NCAMs). The TAM receptors share the same ligand, Gas6, the product of the *growth arrest-specific gene 6* [5], [6]. Gas6, cloned from serum-starved fibroblasts, is a member of the vitamin K-dependent family of Gla proteins homologous to the blood coagulation protein S [7]. However, Gas6 is not believed to harbor any coagulation properties [8], [9]. Instead, Gas6 in general serve as a mitogen and survival factor that protects cells from serum starvation-induced apoptosis [10].

Adequate evidence supports the role of the Gas6/Axl system in driving cell growth and survival in normal and cancer cells [4]. Axl was originally cloned as a transforming gene in human chronic myeloid leukemia and myeloproliferative disease [11], [12], and shown to transform NIH3T3 cells and render them tumorigenic *in vivo* [12]. Since then, Axl overexpression and signaling has been implicated in several human malignancies, such as colon [13], breast [14], glioma [15], thyroid [16], gastric [17], melanoma [18], lung cancer [19], and in renal cell carcinoma (RCC) [20]. A more detailed role of Axl biology has been proven in glioma, where loss of Axl signaling diminished glioma tumor growth [21], and in breast cancer, where Axl drive cell migration, tube formation, neovascularization, and tumor growth [22].

Recently, we have seen that Axl and Gas6 expression correlates to survival in a large cohort of RCC patients [20]. Interestingly, we found low Axl mRNA to independently correlate with substantially longer patient survival. Furthermore, patients with low Axl and high Gas6 mRNA levels as a combination had further increased survival.

RCC is a common tumor of the kidney with poor prognosis. Several RCC tumor types are present based on different histopathological and specific genetic variations. Clear cell RCC (ccRCC) represents the majority of RCCs and alteration in the *von Hippel-Lindau* (VHL) tumor suppressor gene is one of the most common genetic alterations occurring in about 80% of these cases. The VHL protein function as an ubiquitin ligase and targets the hypoxia inducible transcription factors HIF-1α and HIF-2α for degradation. Loss of VHL function leads to a pseudo-hypoxic response in ccRCC that conveys much of the disease progression. [23], [24]

In the kidney, Gas6 and Axl are mainly expressed in glomeruli, tubular endothelial and mesangial cells [20], [25], [26] and Gas6 functions as an Axl-specific autocrine growth factor in mesangial cells [27]–[29].

In the present investigation, we used a cell based RCC model system in order to explore the complex role of Gas6 and Axl in RCC. Our results contribute to the understanding of the multifaceted molecular biology of the disease.

## Results

### Active Axl/Gas6 System in ccRCC Tissue Biopsies

**Figure 1:**
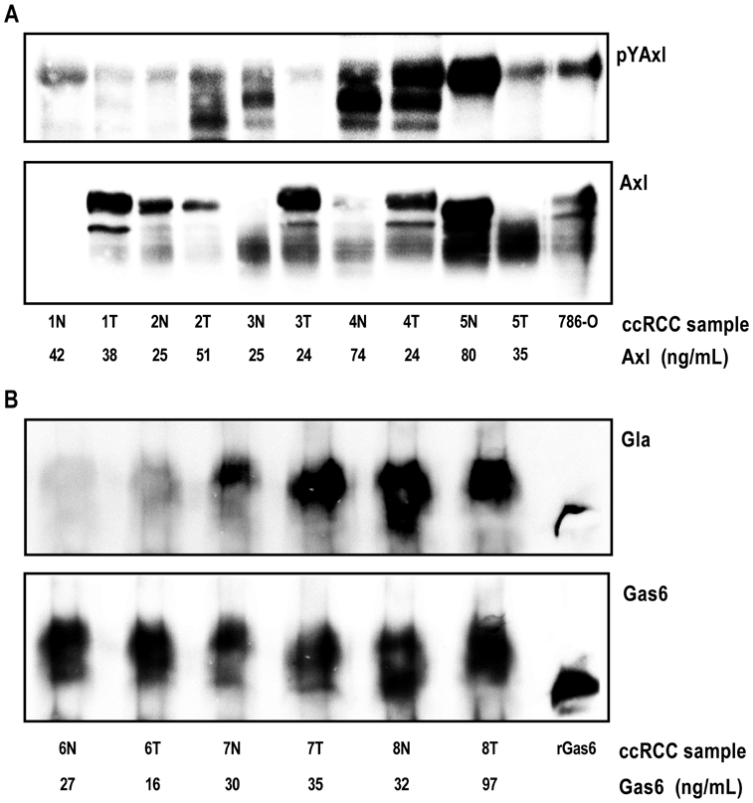
Axl is phosphorylated and Gas6 is γ-carboxylated in human ccRCC tissue. Homogenized tissue lysates from a panel (patient one to eight) of matched ccRCC tissues and unaffected kidney cortex counterparts were used for Axl (*A*) and Gas6 (*B*) immunoprecipitation with specific polyclonal antisera (#R042 towards Axl N-terminal; #005 towards Gas6) containing 50–100 µg total immunoglobulin molecules (about 0.5–1.0 µg specific immunoprecipitating antibody). Pellets were separated on a 6% denaturing SDS-PAGE gel. Protein expression and posttranslational modifications were verified with western blot analysis using Axl (Axl-C20; towards Axl C-terminal), Gas6 (R&D), phosphotyrosine (PY99) and Gla (M3b; 5 mM EDTA final) specific antibodies, respectively. Membranes were first immunoblotted for phosphotyrosine and Gla, respectively, and thereafter stripped and reprobed for total Axl and Gas6. For each patient the matched samples are denoted: N (normal unaffected kidney cortex) and T (ccRCC tissue). Absolute concentrations in the lysates measured by ELISA are depicted below each sample.

Axl was present in homogenized tissue lysates from a panel of matched ccRCC patient biopsies and their respective unaffected kidney cortex tissue counterparts (Fig. 1*A*). Since Axl phosphorylation and activation occurs upon binding of its ligand Gas6 [30], we investigated the phosphorylation status of Axl in the biopsies. All three known Axl glycosylation isoforms (140, 120, and 104 kDa) [11], [31] were phosphorylated with various intensities in human ccRCC tissue (Fig. 1*A*; upper panel). Interestingly, compared to total amount of Axl protein immunoprecipitated (Fig. 1*A*; lower panel), the level of phosphorylation in the different biopsies varied extensively. Some biopsies with low Axl protein had very high phosphorylation status, e.g., lane seven, and vice versa, some biopsies with high Axl protein expression showed no or low phosphorylation level, e.g., lane two, three, and six (Fig. 1*A*).

Moreover, since Gas6 ligand only is functional and can stimulate Axl properly when correctly γ-carboxylated processed by Vitamin K [32], we examined whether Gas6 was present and γ-carboxylated in the matched ccRCC biopsies. We found Gas6 to be expressed and correctly γ-carboxylated *in vivo* (Fig. 1*B*).

We also measured absolute expression levels of Axl and Gas6 in the different biopsies using specific ELISA assay, and the concentrations are given below each figure (Fig. 1*A,B*). Since the immunoprecipitating antibody was the limiting step (about 0.5–1 µg specific immunoprecipitating antibody) the various Axl and Gas6 concentrations detected by the ELISA assays could not be reflected by the levels detected on western blot analysis (Fig. 1*A,B*).

### Axl and Gas6 Expression in ccRCC 786-O Cells

**Figure 2:**
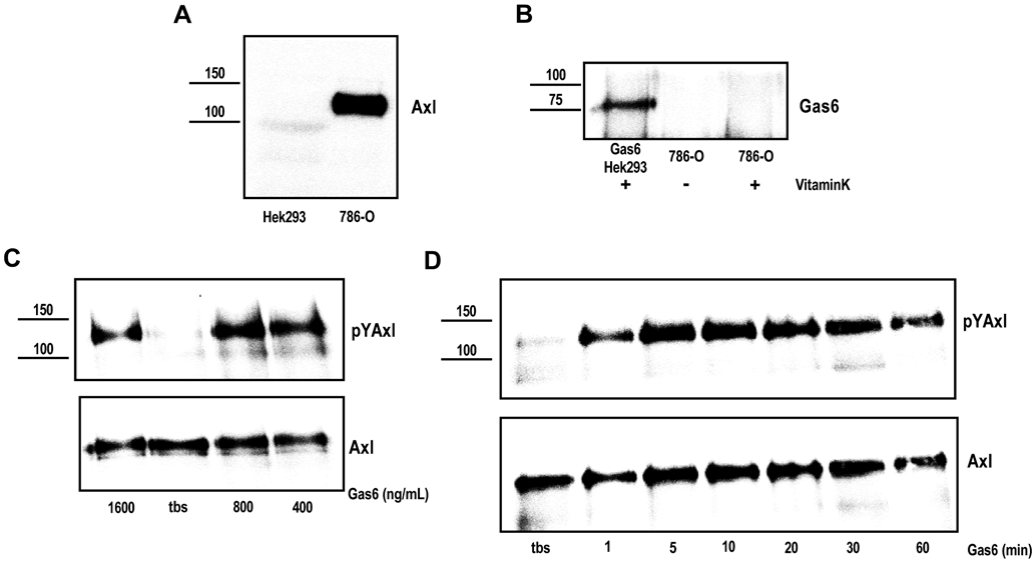
Axl but not Gas6 is expressed in ccRCC 786-O cells and can be functionally active. Expression of Axl (*A*) and Gas6 (*B*) were analyzed by western blot analysis using total cell lysate (tcl; Axl) and immunoprecipitate (Gas6) separated on an 8% reducing SDS-PAGE gel, and using Axl (Axl-C20) and Gas6 (R&D) specific antibodies, respectively. Gas6 expression in 786-O cells, grown in conditioned medium with and without vitamin K, was compared to expression in stably transfected Hek293 cells [51]. Gas6-dependent Axl phosphorylation was analysed in serum-starved (1% FCS; 20 h) 786-O cells stimulated with various Gas6 concentrations for 10 min (*C*) and stimulated with 400 ng/mL Gas6 for up to 1 h (*D*). Total cell lysate of stimulated cells were subjected to Axl immunoprecipitation with an in house Axl-specific polyclonal antibody (R042) and immune-complexes were thereafter separated on 8% reducing SDS-PAGE and immunoblotted for phosphorylated Axl (PY99). The membranes were stripped and reblotted for total amount of immunoprecipitated Axl (Axl-C20). Representative western blots are shown.

In our attempt to study the biological role of Gas6 and Axl signaling in a cell based model system, we first verified the expression and functionality of Axl and Gas6 in the ccRCC 786-O cell line deficient in the VHL tumor suppressor protein. Western blot analysis showed high expression levels of Axl protein in these cells (Fig. 2*A*). However, no Gas6 protein could be found neither with western blot on total cell lysate (data not shown) nor with immunoprecipitation (Fig. 2*B*). In contrast to the results obtained using western blotting, a weak expression of Gas6 mRNA in the same range as that of Gas6 mRNA in ccRCC tumor could be detected using quantitative real-time PCR (qRT-PCR; Table 1). The quantity of Axl mRNA in 786-O cells was very high relative to Gas6 mRNA levels and relative to Axl mRNA levels in the pair of matched normal and tumor RCC tissues (Table 1). The relative expression of Axl mRNA was as high as seen in the limited number of ccRCC tumors analyzed in our previous study [20]. By employing VHL reconstituted 786-O cells we were able to clarify the impact of functional VHL on Axl and Gas6 expression. No difference in mRNA levels could be detected in the three 786-O subclones (786-O wt, mock, and VHL reconstituted; Table 1).

**Table 1:** Gas6 and Axl mRNA expression in a ccRCC kidney and cell line.

Relative mRNA expressionξ					
	786-O wt	786-O mock	786-O VHL	RCC tumor	RCC normal
Axl	0.962	1.307	1.047	0.270	0.104
Gas6	0.0011	0.0022	0.0011	0.0002	0.0007

ξ qRT-PCR measurement relative to human β2-microglobulin reference gene.

786-O wt, mock and VHL refers to ccRCC 786-O cells that are not transfected (defective VHL), empty vector transfected (defective VHL) and VHL reconstituted (functional VHL), respectively.

### Gas6 Induces Axl Phosphorylation in ccRCC 786-O Cells

In order to verify functionality of our model system, the ability of Gas6 to bind and activate Axl was determined by evaluating the ligand-dependent phosphorylation of Axl in 786-O cells. Gas6 stimulated cells showed phosphorylated Axl, while TBS control treated cells show no or markedly low basal Axl phosphorylation levels (Fig. 2*C,D*). Using 400 ng/mL of Gas6 yielded saturated Axl phosphorylation levels (compared to higher concentrations of Gas6; Fig. 2*C*). Lower doses of Gas6 stimulation resulted in Axl phosphorylation displaying a dose-response relationship (data not shown). Gas6 stimulation timecourse revealed a peak of phosphorylated Axl levels between 5 and 10 minutes (Fig. 2*D*). Notably, there was a 2-fold higher induction level of Gas6-dependent Axl phosphorylation in cells grown in 1% FCS compared to cells grown in 10% FCS (data not shown). Furthermore, we found a modest increase in phosphorylated Erk levels after 10 min of Gas6 stimulation (Figure S1 *B*). No influence of Gas6 stimulation on Akt phosphorylation could be found (Figure S1 *A*). Intriguingly, our preliminary results suggested a dip in total Akt levels at 20 min of Gas6 stimulation, which was a consistent observation that we currently cannot explain (Figure S1 *A*).

### Gas6 Inhibits Migration and Cell-Viability of ccRCC 786-O Cells Mediated through Axl

#### Migration

**Figure 3:**
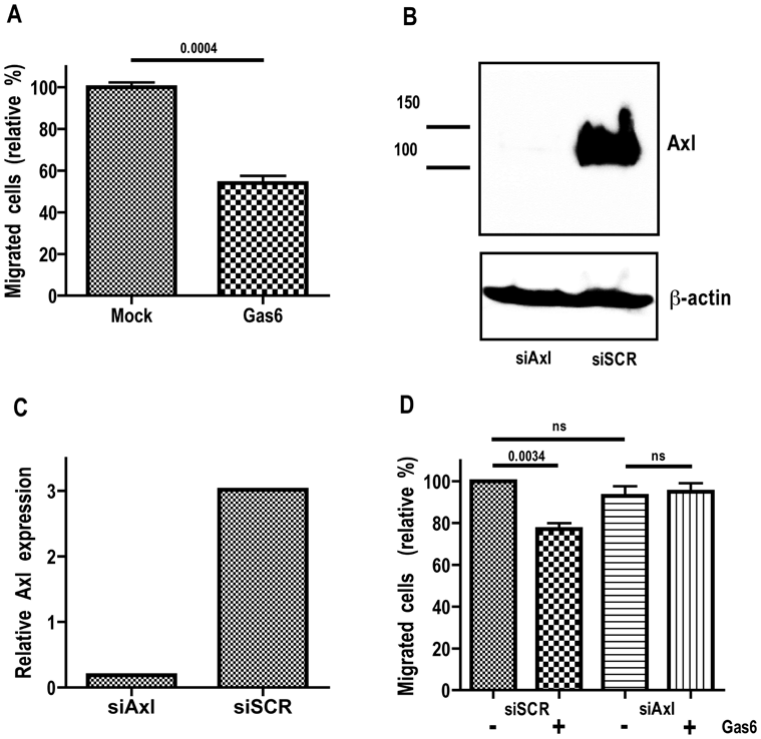
Gas6-dependent Axl-mediated inhibition of ccRCC 786-O cell migration. The migratory capacity of 786-O cells upon Gas6 stimulation was analyzed using the Boyden chamber assay. (*A*) Gas6 stimulation (400 ng/mL) for 4 h decreases the migratory capacity of 786-O cells to about 80% in comparison to that of mock treated cells. Verification of the Axl knockdown using siAxl and siSCR treated cells by either (*B*) western blot analysis or (*C*) qRT-PCR as described in material and methods. (*D*) The inhibition of migration due to Gas6 stimulation was a consequence of ligand specific activation of the Axl receptor as confirmed by repeating the migration experiment using the siAxl and siSCR treated 786-O cells. Each experiment was conducted in triplicates, and the result out of three independent experiments is shown. Fig. 3 *B,C* shows results from one representative experiment.

To examine the effect of Gas6 stimulation on 786-O cell migration, the chemotaxis-based Boyden chamber migration assay was employed. A significant decrease in migration was seen after 4 h of stimulation with 400 ng/mL Gas6 compared to mock stimulation (Fig. 3*A*).

In order to verify that the Gas6-dependent decrease of migration was due to specific ligation and activation of the Axl receptor by Gas6 we performed the migration experiment using Axl knockdown cells. The abrogation of the Axl protein expression was performed using Axl silencing RNA (siAxl). Axl protein expression was undetectable by western blot analysis after silencing compared to control cells transfected with scrambled siRNA (siSCR; Fig. 3*B*). The mRNA levels of the Axl transcript was decreased to approximately 7% of that of control transfected cells (Fig. 3*C*). Gas6 stimulation (400 ng/mL) of Axl-knockdown cells yielded no significant difference in migratory capacity compared to mock-stimulated siAxl cells (Fig. 3*D*), verifying that the Gas6-dependent inhibitory effect was mediated through the Axl receptor and that Axl *per se* does not contribute to the migratory capacity of 786-O cells (Fig. 3*D*).

#### Viability

**Figure 4:**
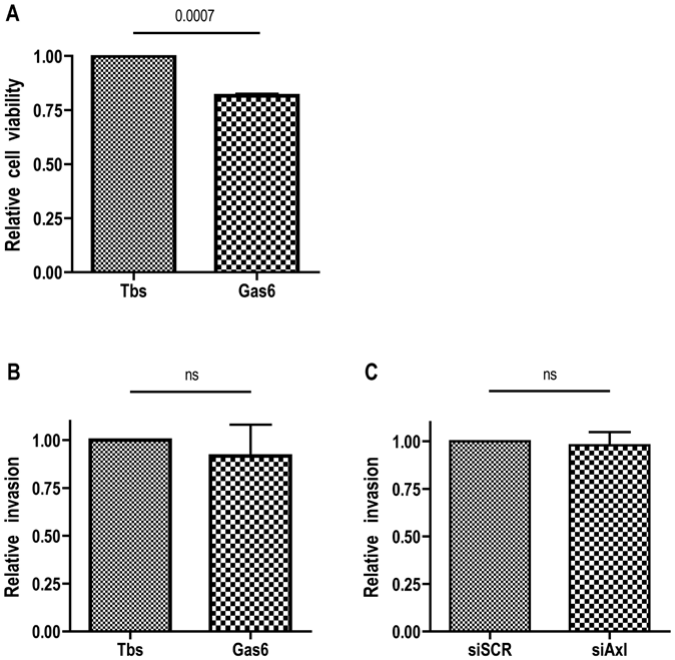
Gas6-dependent Axl-mediated effects on ccRCC 786-O cell viability and invasion. Viability of 786-O cells were analysed using the MTT assay. (*A*) Stimulation of 786-O cells with 400 ng/mL Gas6 for 24 h resulted in a viability of about 80% in comparison to that of control treated cells. (*B*) Invasion of 786-O cells using the modified Boyden chamber assay with a matrigel layer for analysis of invasive capacity was not affected by Gas6 stimulation (400 ng/mL). (*C*) Axl expression *per se* did not contribute to the high invasive phenotype of 786-O cells, since the lack of Axl expression did not result in any significant diminished invasive capacity. Each experiment was performed using at least triplicates and the result is shown from three independent experiments.

Using the tetrazole 3-(4,5-Dimethylthiazol-2-yl)-2,5-diphenyltetrazolium bromide (MTT) assay, we examined the metabolic activity as an estimation of 786-O cell-viability after stimulation with Gas6. A modest but clear decrease in viability was seen after 24 h of Gas6 stimulation (400 ng/mL) compared to TBS control stimulated cells (Fig. 4*A*). Knockdown of Axl expression did not by itself affect the viability of 786-O cells (data not shown).

### Invasiveness of ccRCC 786-O Is Independent of the Gas6/Axl System

We wanted to investigate the role of Axl in the mechanism of invasion of 786-O cells, known to have high invasive potency. For this purpose, we used the modified Boyden chamber assay in which the cells have to invade a matrigel layer. We could not detect any effect of Gas6 stimulation on the invasive phenotype of 786-O cells in comparison to TBS control (Fig. 4*B*). Since it is previously shown that Axl mediates invasion both in a Gas6-dependent [17], [21] and independent manner [33], we investigated the invasive phenotype of Axl knockdown 786-O cells. However, we could not detect any significant decrease of invasion of cells where Axl RTK expression was silenced compared to normal cells (Fig. 4*C*).

### Regulation of Axl Protein Expression in ccRCC 786-O Cells

#### Gas6-dependent Axl downregulation

**Figure 5:**
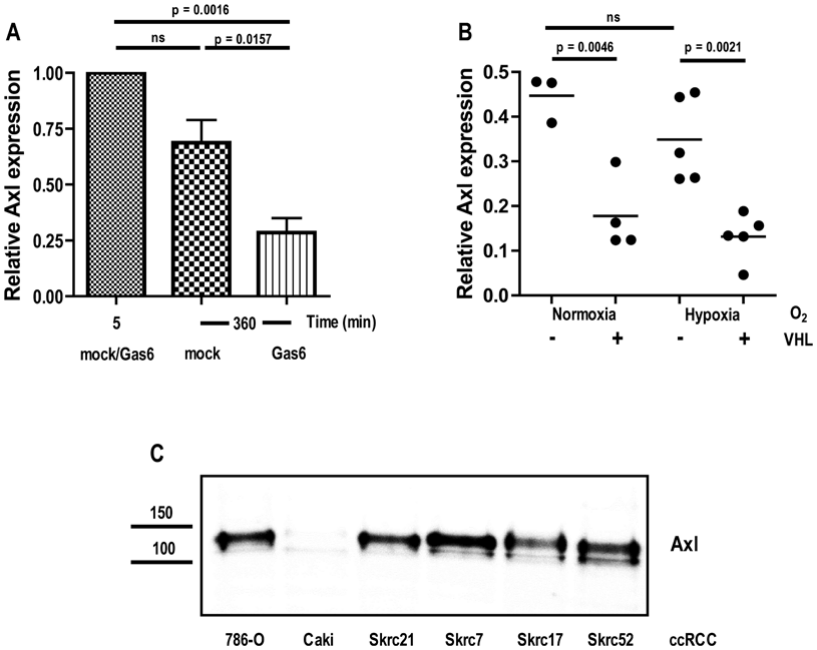
Gas6- and VHL-dependent Axl protein expression in ccRCC 786-O cells. (*A*) Serum-starved 786-O cells (1% FCS; 20 h) were stimulated with 400 ng/mL Gas6 or mock control for up to 6 h. Gas6-dependendent Axl protein down-regulation over time were verified with western blot analysis on total cell lysate from treated cells using 8% reducing SDS-PAGE. Membranes were immunoblotted with Axl specific antibodies (Axl-C20) and reprobed with β-actin specific antibodies (A5441) for relative quantification of Axl expression levels. Each experiment was run in duplicates and the result of three independent experiments is shown. (*B*) Axl expression in 786-O mock (VHL defective) or VHL reconstituted cells was analyzed in cells grown in either normoxia or hypoxia (1% O_2_) under serum starvation (1% FCS) for 48 h. VHL-dependent Axl expression was verified using western blot analysis of total cell lysates of treated cells that were separated on an 8% reducing SDS-PAGE gel. Membranes were immunoblotted with Axl-specific antibodies (Axl-C20) and with β-actin specific antibodies (A5441) for relative quantification of Axl expression levels. The experiment was carried out using a minimum of triplicates. (*C*) Axl inverse correlation to functional VHL protein was further analyzed by immunoblotting for Axl expression in total cell lysates of a panel of ccRCC cell lines with different VHL status. Equal amounts of total protein were separated on an 8% reducing SDS-PAGE gel and membrane immunoblotted with Axl-specific antibodies (Axl-C20).

Stimulation of 786-O cells with 400 ng/mL Gas6 for up to 6 h resulted in a time-dependent down-regulation of Axl protein. After 6 h of Gas6 stimulation, the expression of Axl protein was almost undetectable by western blot analysis (data not shown). The amount of Axl protein was significantly decreased after 6 h of stimulation compared to the first time-point and compared to mock stimulated cells (Fig. 5*A*).

#### Axl protein level is inversely related to VHL expression

As shown in Table 2 Axl mRNA levels was not affected by introduction of wt VHL in the VHL deficient 786-O cells. In contrast, when Axl protein levels were assessed in the 786-O subclones a considerable difference was detected. Axl protein was decreased to about half in VHL reconstituted cells compared to mock transfected or untransfected VHL defective cells. Importantly, the Axl protein levels remained low also when the VHL reconstituted cells were grown at hypoxia (1% oxygen) and hence reactivate the hypoxic response due to stabilization of HIF-2a (Fig. 5*B*). These results suggest that Axl is not a direct HIF-2α gene target (HIF-1α is not expressed in 786-O cells [34], [35]) Moreover, Axl protein expression was high in ccRCC cell lines with defective VHL protein compared to Caki-2 [36], a ccRCC cell line with papillary RCC characteristic and functional VHL (Fig. 5*C*). Together, these results show that loss of VHL is associated with elevated levels of Axl through a yet unknown post-transcriptional mechanism.

**Table 2:** ccRCC tumor characteristics at time of nephrectomy and patient clinicopathological information.

Patient	Sex (Age)	TNM stageξ	Gradeψ	Survival (months)	Endpoint event
1 (86∶05)	M (77)	3	3	18	Death by disease
2 (88∶12)	F (56)	4	4	12	Death by disease
3 (87∶22)	M (42)	4	2	249	Alive with no signs of disease
4 (88.02)	F (55)	4	2	37	Death by disease
5 (86∶10)	M (75)	1	3	33	Death by disease
6 (87∶24)	F (82)	3	3	103	Death by disease
7 (87∶26)	F (53)	1	2	245	Alive with no signs of disease
8 (08∶07)	F (77)	4	4	8	Death by disease

ξ According to the tumor-node-metastasis (TNM) classification system 2002 [53].

ψ According to Skinner nuclear grade [54].

## Discussion

We demonstrate inhibition of migration and viability in ccRCC 786-O cells as a result of Gas6 signaling through the Axl RTK. However, the highly invasive phenotype of these cells was not affected by the Gas6/Axl system. So far, in most human cancers where the biology of Gas6 and Axl has been studied, Axl is believed to exert oncogenic effects [13]–[16], [19]–[21], [37], [38], and a combined increase of Gas6 and Axl expression correlate with poorer prognosis [15], [37]. Increased Gas6 expression correlating with favorable prognosis has so far only been reported in human breast cancer [39] and in RCC [20], which partly may be explained by mechanisms presented here.

Our results are in large contradictory to the common picture of Gas6 and Axl signaling. Gas6 has for instance been shown to induce chemotactic migration [40]–[42], to be an autocrine growth factor in glomerular mesangial cells [28], [29], and to drive glomerular hypertrophy [43]. Invasiveness has been correlated with the expression of Axl [19], [38], and Gas6-dependent signaling through Axl has been shown to promote invasion of glioma cells [21]. However, in line with our results, Gas6 has also been shown to have opposing effects by inhibiting VEGFR-A driven migration specifically through the activation of Axl RTK [44].

Our results together with known literature imply that the biology of Gas6 and Axl is complex and probably context-specific. Perhaps in RCC, where high Gas6 expression correlate with improved prognosis [20], Gas6 can be protective and retard cancer progression by mechanisms, such as decreased migratory potential and decreased viability. High Axl mRNA expression is in itself negative for the outcome of RCC patients [20], and prolonged Gas6 stimulation of 786-O cells leads to protein down-regulation of Axl. However, our present data do not provide an explanation how Axl by itself contribute to poor prognosis. It is possible that the adhesion-like extra cellular region of the Axl receptor with its ability to mediate homophilic interactions and adhesion [45], [46] can contribute to a more aggressive cancer phenotype *in vivo* independent of Gas6 stimulation. Other putative uncharacterized contributions of Axl in cancer progression can also be discussed. For instance, Axl dependent signal transduction and survival mediated by other heterophilic interactions has been described [47].

Another key aspect of the pathogenesis of ccRCC is the loss of expression of the tumor suppressor VHL protein resulting in pro-angiogenic stimulus of the cancer cells due to increased expression of VEGF and autocrine signaling. [24] Interestingly, we found an association between expression of Axl protein and the tumor suppressor VHL in 786-O cells. When a functional VHL protein was introduced, Axl protein levels decreased to about half. No difference could be found in Axl mRNA levels. Furthermore, Axl is not a gene target of HIF-2α, since there was a VHL dependent downregulation of Axl in cells grown in hypoxia as well. As mentioned, Axl has been shown to affect neovascularization *in vitro*, and loss of Axl expression in tumor cells blocks growth of human neoplasms [22]. Perhaps, Axl on its own, by homophilic interactions and by a kinase domain-dependent mechanism [45], contribute to the disease-specific angiogenic program during VHL loss in tumor cells in parallel with angiogenic factors such as VEGF. Gas6 signaling via Axl on the other hand has been shown to have inhibitory effects on the VEGFR-driven angiogenic program [44]. One might therefore speculate that at high Axl expression levels, increased Gas6 signaling through Axl could be anti-angiogenic and beneficial. These interesting results indicate that there is a need for further exploration of the role of Axl and Gas6 in regulation of the angiogenic process in ccRCC and moreover, to determine whether VHL function as an Axl-specific ubiquitin ligase or regulates Axl protein expression level by another unknown mechanism.

Our results demonstrate that the Gas6/Axl system plays a role in RCC, and that Gas6 not always functions as a mitogenic growth factor. Our results lay the ground for further investigation of the complex biology of Axl and Gas6 in RCC and several other diseases where this signaling pathway contribute to the pathological process.

## Materials and Methods

### Patient Biopsies

Tumor biopsies and normal kidney cortex tissues were taken from ccRCC patients. The study was approved by the ethical committee of Umeå University and by the Institutional Review Board. Each patient participated after providing informed written consent. For detailed information see [20]. Clinicopathological characteristics of patients and tumors are shown in Table 2.

### Cell Culture and Reagents

The ccRCC cell line 786-O wt [48] (American Type Culture Collection; LGC Standards AB) was maintained in DMEM supplemented with 10% FCS and 1% penicillin and streptomycin and 1% L-glutamine. The mock (pRC3) and VHL reconstituted (WT-7) 786-O cell lines [49] were generous gifts from W.G. Kaelin Jr. (Dana Farber Cancer Institute) and were maintained in the same medium supplemented with the selective agent G418 (0.8 mg/mL Geneticin; Roche Applied Science, Life Technologies).

For evaluation of Gas6 expression in 786-O wt cells, cells were grown in supplement of 10 µg/mL vitamin K (Konakion; Roche). The ccRCC Caki-2 cell line [36], and the ccRCC SKRC -7, -17, -21 (kindly provided by Dr. E. Oosterwijk (Radboud University Mijmegen medical Center, The Netherlands), and SKRC-52 cell lines [50] were maintained in the same medium as 786-O cells.

In hypoxia experiments, cells were grown in 1% oxygen in a 400 Hypoxia Workstation (Ruskinn Technology Ltd.; MORTEK AS) connected to a Fuskinn gas mixer module.

### Preparation of Recombinant Gas6

Recombinant human Gas6 was expressed and collected in serum-free OPTIMEM as described previously [51]. Monoclonal antibody affinity purified Gas6 was thereafter obtained (to be described elsewhere). In experiments where Gas6 control is designated mock, Gas6 in OPTIMEM was used for Gas6 stimulation (Gas6), and OPTIMEM harvested from mock-transfected cells as control (mock). In experiments where Gas6 control is designated TBS, purified Gas6 was used for Gas6 stimulation (Gas6), and purification buffer as control (TBS).

### Preparation of ccRCC Lysates

Removal of ccRCC patient biopsies was performed as described [20]. To extract total protein, 200 mg of frozen biopsies were crushed with a mortar and pestle in liquid nitrogen. Powdered tissues were directly dissolved in ice-cold lysis buffer (1% Triton X-100, 25 mM Tris-HCl pH 7.5, 150 mM NaCl, 5 mM EDTA, and 10% glycerol supplemented with 100 mM PMSF, 200 mM Sodium Orthovanadate and the protease inhibitor cocktail set III (#539134, Calbiochem, MERCK; according to manufacturer) containing AEBSF-Hydrochloride, Aprotinin, Bestatin, E-64, Leupeptin and Pepstatin A. Suspensions were then vortexed for 1 min and thereafter incubated on ice for 45 min. Finally, the lysed suspensions were homogenized with a polytron (IKA T 8 Ultra-Turrax, IKA) at level 5 for 2×15 s, centrifugated at 20 000× *g* at 4°C for 5 min, and clear supernatants were transferred to clean tubes for storage on ice until analysis the same day (2/3 of sample was used for immunoprecipitation and the remaining sample was used for the ELISA assays).

Preparation of lysate from ccRCC cell lines was performed as follows. Adherent cells were put on ice. Medium was removed and cells were washed once in ice-cold PBS. Thereafter, ice-cold lysis buffer (1% Triton X-100, 25 mM Tris-HCl pH 7.5, 150 mM NaCl, 5 mM EDTA, and 10% glycerol supplemented with 100 mM PMSF, 200 mM Sodium Orthovanadate, and 200 µg/mL Aprotinin) was added to the plate, and lysis was allowed for 20 min with agitation at 4°C. After centrifugation at 20 000× *g* for 1 min at 4°C, the clear supernatants were transferred to fresh tubes and stored on ice until use on the same day for immunoprecipitation and western blot analysis.

### Immunoprecipitation

Lysates (volume of ccRCC tissue lysates was 500 µL respectively) were pre-cleared with 10 µL pre-immune sera for 30 min at 4°C with rotation followed by incubation with 10 x excess protein A-Sepharose (in relation to immunoglobulin G-binding capacity; #10-1041, Invitrogen Corporation) for another 30 min. Immune complexes were removed by centrifugation at 20 000× *g* for 1 min at 4°C and the pre-cleared lysates were subsequently incubated with polyclonal antisera containing 50–100 µg total immunoglobulin molecules (about 0.5–1 µg of specific immunoprecipitating antibody) for 1 h at 4°C with rotation followed by incubation with 10 x excess protein A-Sepharose for another 30 min. Immune complexes were pelleted by centrifugation at 20 000× *g* for 1 min at 4°C, and washed x 2 in ice-cold lysis buffer and finally x 1 in ice-cold TBS-T (50 mM Tris-HCl pH 7.4, 150 mM NaCl and 0.1% Tween 20). The immunoprecipitated protein was released by dissolving the pellet in reducing SDS-PAGE sample buffer (200 mM Tris-HCl pH 8.8, 60% sucrose, and Bromophenol blue supplemented with dithiothreitol (DTT) to 10 mM final sample concentration).

Antibodies used to immunoprecipitate Axl and Gas6 from ccRCC total cell lysate or from human ccRCC biopsies, were generated as rabbit polyclonal anti-Axl (Ig-fraction of an in-house polyclonal rabbit antiserum denoted R042; towards Axl N-terminal) or anti-Gas6 antibodies (Ig-fraction of an in-house polyclonal rabbit antiserum denoted R05).

### Western Blot Analysis

Protein samples were separated on an 8% SDS-PAGE gel and the proteins were transferred from the gel onto a polyvinylidene fluoride (PVDF) membrane (Pall Corporation; VWR International AB). After blocking, membranes were incubated with the following unlabeled primary antibody: anti-phosphotyrosine (pY99; sc-7020, Santa Cruz Biotechnology), anti-Axl (C-20; towards Axl C-terminal; sc-1096, Santa Cruz Biotechnology), anti-Axl (#AF154; towards Axl N-terminal, R&D Systems), anti-Gas6 (AB885, R&D Systems), anti-γ-carboxyglutamyl acid (anti-Gla; M3b) [52], *kind gift from Dr. Johan Stenflo*), anti-β-actin (A5441, Sigma-Aldrich). HRP-conjugated secondary antibodies were obtained from DAKO. Chemiluminescence was developed with Immobilon Western Chemiluminescent HRP Substrate (WBKL S0500, Millipore) and traced with Fuji LAS 3000IR CDD camera, and the signals were quantified with the Image Gauge program (FUJIFILM, Science Lab 2003, version 4.1).

### Quantitative Real-Time Reverse Transcription PCR

One-step multiplex quantitative real-time reverse transcription PCR (qRT-PCR) was employed using primary tumor total RNA and matched histopathologically non-malignant kidney cortex tissue serving as a reference (commercial ccRCC tumor RNA of nuclear grade 3; Ambion). The qRT-PCR analysis was employed as described previously [20].

### ELISA Assay for the Quantification of Axl and Gas6

Concentrations of sAxl and Gas6 protein ccRCC patient material and their matched normal counterparts were estimated using in-house developed sandwich ELISA assays specific for sAxl and Gas6 as previously described [20].

### siRNA Transfection

ccRCC 786-O cells were seeded in 60 mm plates 24 h preceding transfection. Thereafter, cells were transfected with siRNA targeting Axl (siAxl; sc-29769, Santa Cruz Biotechnology) or scrambled control siRNA (siSCR; sc-37007, Santa Cruz Biotechnology) using Lipofectamine*™*2000 (#11668-019, Invitrogen) in serum-free OPTIMEM (GIBCO, Invitrogen) for 5 h at 37°C. The medium was then replaced with conditioned DMEM containing 10% FCS and cells incubated for another 24 h. Transfected cells were then used in the Boyden chamber assay (below) to analyze their migratory and invasive capacity.

### Boyden Chamber Migration and Invasion

Boyden chambers with 24-wells (Transwell® Permeable Supports; Cole-Parmer; BergmanLabora AB) were used to evaluate the migratory capacity of Gas6 or mock stimulated wt ccRCC 786-O cells siAxl and siSCR transfected cells. The chambers consist of an upper and lower space separated by a microporous polycarbonate membrane with a pore size of 8 µm. Membranes of each chamber were incubated in serum-free medium for an initial equilibrium period of 1 h. A cell concentration of 1×10^6^ cells/mL was added to each migration (upper) chamber. Cells were allowed to attach for 2 h and thereafter chemotaxis was induced by addition of 10% FCS to the lower chamber. Simultaneously, 400 ng/mL Gas6 (final concentration) or mock control were added to both the upper and lower chamber and the cells were allowed to migrate from the upper compartment through the membrane towards the lower compartment along the chemoattractant gradient.

Migration was allowed for 4 h at 37°C. The chambers were then cleaned with a cotton swap with 1 x PBS to remove the cells left on top of the membranes. Migrated cells were fixed for 15 min in 4% paraformaldehyde. Membranes were cut out with a needle, put on a glass slide and finally the cells were stained with 4′6-diamidino-2-phenylindole (DAPI; Vector Laboratories Inc.; IMMUNKEMI F&D AB). Migrated cells could be counted using microscopy at 40× magnification.

In the invasion assay, Boyden chambers were coated with 50 µL of 12.5% growth factor reduced matrigel (#354230, BD Bioscience) and incubated at 37°C for 1 h. A cell concentration of 1×10^6^ cells/mL was added to each pre-incubated invasion chamber and cells were allowed to attach for 2 h with no chemoattractant. Thereafter, chemotaxis was induced by addition of 10% FCS to the lower chamber. Simultaneously, 400 ng/mL Gas6 (final concentration) or TBS control were added to both the upper and lower chamber and the cells were allowed under 16 h at 37°C to invade the matrigel and migrate. Cells were harvested as described in the migration assay. In both migration and invasion assay three representative fields were counted for each membrane and each treatment condition was assayed in triplicate and repeated three times.

### MTT Assay

ccRCC 786-O cells were seeded in a 96-well plate at a density of 2.5×10^4^ cells/mL for 24 h at 37°C. Thereafter cells were treated with 400 ng/mL Gas6 or TBS control and incubation was continued for another 24 h. Thereafter, MTT dye solution (Promega Corporation; Promega Biotech) was added and incubation continued for another 4 h. The assay was ended by addition of Stop/Lys solution (Promega Corporation) followed by incubation over night in a light-protected container at room temperature. The absorbance generated was read using the ELISA-plate (anthos 2020, anthos labtec instruments) reader at 595 nm using 690 nm as a reference.

### Statistical Analyses

For statistical analysis comparing one condition to a normalized value, the one sample t-test to a theoretical value was used. Otherwise, when comparing different conditions within one experiment the unpaired t-test was employed. Data are presented as the mean ± SD. All statistical tests were two-sided, and the significance level was set at 0.05. Statistical analyses were performed using the GraphPad Prism software package (version 4.0c for Macintosh).

## Supporting Information

Figure S1 Gas6-stimulated ccRCC 786-O cells display a modest increase in phosphorylated Erk levels.
(0.15 MB PDF)
