# Supplementary material for: Gas6 and the Receptor Tyrosine Kinase Axl in Clear Cell Renal Cell Carcinoma
Source: PLoS One. 2009 Oct 30;4(10):e7575. doi: 10.1371/journal.pone.0007575 (PMC2766033; doi:10.1371/journal.pone.0007575)
Supplement: Figure S1 — Gas6-stimulated ccRCC 786-O cells display a modest increase in phosphorylated Erk levels. (0.15 MB PDF) [file pone.0007575.s001.pdf]

Supplementary information

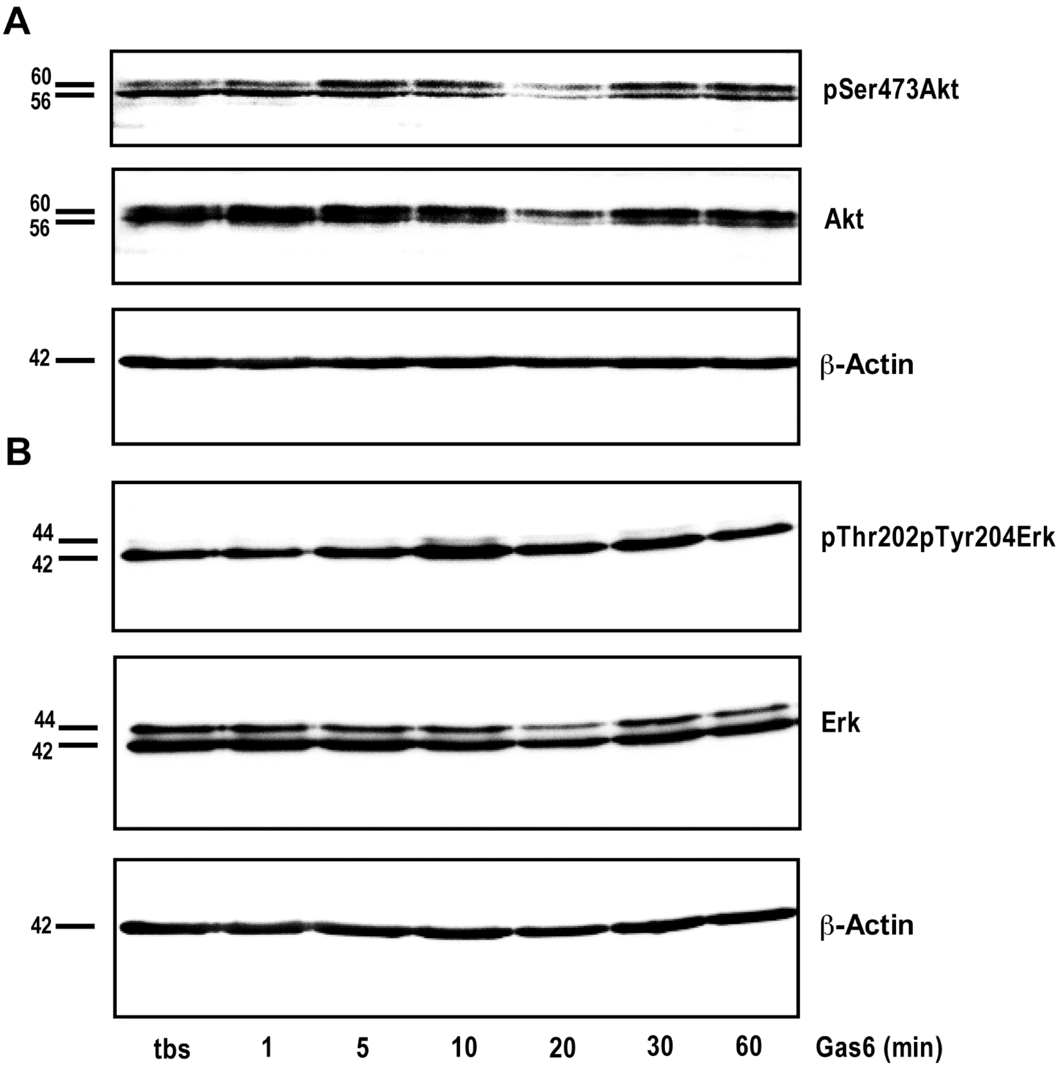

Figure S1. Gas6-stimulated ccRCC 786-O cells display a modest increase in phosphorylated Erk levels.

786-O cells were stimulated with 400 ng/mL Gas6 for up to 1 h, and Gas6-dependent Axl phosphorylation was verified (Figure 2*D*). Expression of phosphorylated Akt/total Akt (*A*) and phosphorylated Erk/total Erk (*B*) were analyzed by western blot analysis as described in material and methods using total cell lysate separated on an 8 % reducing SDS-PAGE gel. Primary antibodies used were specific for phosphorylated Akt at serine 473 (polyclonal

rabbit anti-pSer473Akt; sc-7985), for phosphorylated Erk at threonine 202 and tyrosine 204 (polyclonal rabbit ant-pThr202,Tyr204Erk; sc-16982-R), for Akt 1 and 2 (polyclonal goat anti-Akt; sc-1619), for Erk 1 and 2 (polyclonal rabbit anti-Erk; sc-94), and for β-Actin (monoclonal mouse anti-β-Actin; A5441). Membranes were stripped before reblotting as described in materials and methods. Unlabelled primary antibodies were from Sigma-Aldrich and HRP-conjugated secondary antibodies used for detection were from Dako (described in material and methods). Representative western blots are shown.
